# NEWS AND NOTICES

**Published:** 2015

## Online-only articles

Please visit **www.cehjournal.org** to read the other articles in this issue:

- Planning DR services: step by step
- Research update: DR in sub-Saharan Africa
- DR services in Fiji: attitudes, barriers and screening practices
- A snapshot of DR in Swaziland
- Developing an effective DR screening service
- Empowering patients with DR
- From the field: educating DR patients on gaining better diabetes control
- Online training for DR screening: iTAT

## News and notices

### New South Asia edition of the *Community Eye Health Journal*

We are pleased to announce that the new **South Asia Edition** will soon be available for download to readers in **Bangladesh, Bhutan, Maldives, Myanmar, Nepal, Pakistan** and **India.**

**IMPORTANT** – to ensure you are informed when new issues are published, please send your email address to Shivani Mathur at **editor@cehjsouthasia.org**

The International Edition will continue to be available online at **www.cehjournal.org** but paper copies will no longer be distributed to readers in these countries.

If your educational or training institution requires paper copies, please call +9140 4900 6000, write to the Indian Institute of Public Health, Plot # 1, Rd Number 44, Kavuri Hills, Madhapur, Hyderabad, Telangana 500033, or email **editor@cehjsouthasia.org**

## Teaching institutions

### German Jordanian University

Email: **vtc@gju.edu.jo**

### Community Eye Health Institute

**www.health.uct.ac.za** or email **chervon.vanderross@uct.ac.za**

### Lions Medical Training Centre

Write to the Training Coordinator, Lions Medical Training Centre, Lions SightFirst Eye Hospital, PO Box 66576-00800, Nairobi, Kenya. Tel: +254 20 418 32 39

### Kilimanjaro Centre for Community Ophthalmology International

Visit **www.kcco.net** or contact Genes Mng'anga at **genes@kcco.net**

## Subscriptions


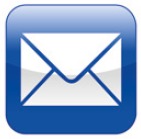
 Contact Anita Shah **admin@cehjournal.org**

### Subscribe to our mailing list

**web@cehjournal.org** or visit **www.cehjournal.org/subscribe**

**Visit us online: www.cehjournal.orgwww.facebook.com/CEHJournal/https://twitter.com/CEHJournal**

